# The Human Archaeome: Commensals, Opportunists, or Emerging Pathogens?

**DOI:** 10.3390/pathogens14111111

**Published:** 2025-10-31

**Authors:** Douglas M. Ruden

**Affiliations:** Department of Obstetrics and Gynecology, Charles Stewart Mott Center for Human Growth and Development, Institute of Environmental Health Sciences, Wayne State University, Detroit, MI 48302, USA; douglasr@wayne.edu; Tel.: +1-313-577-6688

**Keywords:** emerging pathogens, methanogens, archaea and disease, archaeal microbiome, pathogenesis, inflammatory bowel disease, periodontal disease, obesity, microbial interactions, bacteria-MERFISH

## Abstract

Archaea, one of the three domains of life, are increasingly recognized as consistent, though often underappreciated, members of the human microbiome, yet their roles in health and disease remain poorly understood. Unlike bacteria, no archaeal species have been conclusively identified as primary mammalian pathogens, but their widespread presence across diverse body sites suggests potential indirect contributions to host physiology and pathology. Current evidence is synthesized on archaeal diversity and habitat specificity across multiple human-associated sites, encompassing the gastrointestinal, aerodigestive, and urogenital tracts as well as the skin. Methanogens dominate the lower gastrointestinal tract (LGT), where they influence fermentation dynamics and methane production, while members of the class *Nitrososphaeria* are prevalent on the skin and upper aerodigestive tract (UAT), reflecting ecological specialization. Variability in archaeal composition across niches highlights possible links to disease processes: methanogens have been associated with irritable bowel syndrome (IBS), inflammatory bowel disease (IBD), obesity, and colorectal cancer (CRC); *Methanobrevibacter oralis* is enriched in periodontal disease; and archaea have been detected in the lungs of cystic fibrosis patients. Although archaea lack canonical bacterial virulence factors, they may contribute indirectly through metabolic cross-feeding, immune modulation, synergy in polymicrobial infections, and alteration of host–microbiome network dynamics. This review explores the emerging concept of the human “archaeome”, evaluates current evidence for archaeal involvement in disease, and highlights emerging technologies, such as bacteria-MERFISH and multi-omics profiling, that enable translational applications including microbiome diagnostics, therapeutic targeting, and microbiome engineering.

## 1. Introduction

Archaea, first recognized as a distinct domain of life in the late 1970s by Carl Woese and George Fox [1], have historically received far less attention than bacteria and eukaryotes in the study of human biology [2]. For decades, archaeal species were misclassified as bacteria due to their prokaryotic morphology and inability to be readily cultured under standard laboratory conditions [2]. Advances in molecular biology, particularly ribosomal RNA gene sequencing, have since revealed the unique evolutionary position of archaea as a separate domain [3], yet their roles in mammalian health and disease remain underexplored [4].

Unlike bacteria, which have yielded a long list of established pathogens in humans, no archaeal species has been definitively demonstrated to cause disease in mammals [4]. This absence of direct pathogenicity contrasts with their consistent detection across diverse body sites, raising questions about indirect influences on host physiology and microbial ecology. Methanogenic archaea such as *Methanobrevibacter smithii* and *Methanosphaera stadtmanae* are abundant in the gastrointestinal tract [5,6], while *Methanobrevibacter oralis* is frequently detected in the oral cavity [7,8]. In contrast, members of the class *Nitrososphaeria*, which are ammonia-oxidizing *Thaumarchaeota*, are more prevalent on the skin and within the upper aerodigestive tract, where they may influence nitrogen cycling and microbial community dynamics [9,10]. These organisms often coexist with bacteria and may contribute to microbial ecosystem stability. Importantly, their enrichment in certain disease-associated contexts (e.g., periodontal disease, cystic fibrosis, inflammatory bowel disease) has spurred interest in their possible roles as commensals, opportunists, or ecological drivers of pathology.

Several features differentiate archaea from bacteria in ways that may limit their pathogenic potential. Archaea generally lack the virulence factors that are hallmarks of bacterial pathogens, including pore-forming toxins, Type III and Type IV secretion systems, and specialized adhesins [11]. Their unique membrane lipids, ether-linked rather than ester-linked, and their distinct cell wall structures further insulate them from the horizontal gene transfer events that have spread virulence determinants among bacterial pathogens [12]. For these reasons, archaea are not considered primary pathogens in mammals [12]. However, genomic analyses of human-associated archaea suggest the presence of stress-response genes, adhesin-like proteins, and surface-layer variability that may facilitate interactions with the host or with bacterial partners, pointing to underappreciated mechanisms of ecological adaptation [13,14].

Evidence is mounting that archaea may exert indirect or opportunistic influences on disease processes [4]. For example, in polymicrobial infections such as periodontal disease, methanogens can consume hydrogen, creating metabolic niches that promote the growth of pathogenic bacteria [7,15]. Similarly, archaeal overrepresentation has been reported in patients with inflammatory bowel disease [16,17] and in animal models of obesity [18,19], suggesting potential roles in host–microbe interactions that extend beyond simple commensalism. At the same time, conflicting results and limited cohort sizes highlight the need for more rigorous, mechanistic studies.

The field of human archaeome research is still in its infancy. Many fundamental questions remain unresolved, including whether archaeal diversity in the human microbiome has been fully appreciated, whether some lineages are more closely linked to pathology than others, and whether the evolutionary trajectory of archaea could eventually yield pathogenic potential [4]. Recent discoveries of new strains such as *Methanobrevibacter intestini* [20] and *M. smithii* “GRAZ-2” [21], which, unlike other *M. smithii* strains, can produce formic acid [21], underscore that archaeal diversity in humans is broader than previously appreciated. With the advent of next-generation sequencing, metagenomics, and single-cell approaches, our capacity to identify and study archaea in complex microbial communities has expanded dramatically, opening new avenues for discovery [22].

In this review, we examine current knowledge of the human archaeome, focusing on its presence in health and disease, its potential roles in pathogenesis, and the technological challenges that have limited prior investigation. We also explore possible future research directions aimed at determining whether archaea will remain categorized as harmless commensals or emerge as bona fide contributors to mammalian disease.

## 2. Materials and Methods Used in Studying the Archaeaome

Because the study of archaea in humans is still developing, the methodologies used to investigate their presence, diversity, and potential pathogenic roles require special consideration. Traditional microbiological approaches designed for bacteria are often inadequate, necessitating tailored strategies for archaeal detection, isolation, and functional analysis.

### 2.1. Molecular Detection and Sequencing

The cornerstone of human archaeome research has been culture-independent molecular techniques. Ribosomal RNA gene sequencing, particularly of the 16S rRNA gene, has revealed archaeal lineages in human samples that would otherwise remain undetected [23]. However, archaeal 16S rRNA genes are less represented in universal primer sets, leading to underestimation of archaeal abundance [24]. To address this, archaeal-specific primers and metagenomic sequencing have been increasingly employed, allowing more accurate detection and characterization of archaeal diversity [10,24]. Careful primer design, inclusion of mock communities, and use of negative controls are critical for minimizing amplification bias and contamination. Metatranscriptomics and metaproteomics further provide insight into archaeal metabolic activity in vivo [25]. Where possible, cross-validation across different omics layers (e.g., metagenomics and metatranscriptomics) strengthens confidence in archaeal activity assignments. Spatial transcriptomics approaches have recently been used to characterize the microbiome transcriptome at single-RNA molecule resolution using multiplexed error-robust fluorescence in situ hybridization (MERFISH) [26].

### 2.2. Archaeal Culture Methods

Culturing archaea remains a significant challenge due to their slow growth rates and unique nutritional requirements. Methanogens, the dominant human-associated archaea, require strict anaerobic conditions and specific substrates such as hydrogen and carbon dioxide [6]. Advances in anaerobic culturing techniques, along with custom-designed growth media, have enabled the isolation of *Methanobrevibacter* and *Methanosphaera* species from human samples [6], although many archaeal taxa remain uncultured [27]. Improving culture techniques is essential for functional studies and for testing causative roles in disease models.

### 2.3. In Vitro Co-Culture and Microbial Interaction Studies

Since archaea are rarely found in isolation within human microbiomes, co-culture studies with bacteria provide valuable insights [27]. For example, co-culturing methanogens with sulfate-reducing or fermentative bacteria allows researchers to investigate metabolic cross-feeding and potential synergistic effects on host tissues [27]. Culture attempts should include parallel molecular confirmation (e.g., qPCR or sequencing) to verify archaeal growth, since morphological features alone can be ambiguous. Such experiments are critical for understanding how archaea may indirectly contribute to pathogenic outcomes by enhancing bacterial virulence or altering microbial community dynamics.

### 2.4. Animal Models and Gnotobiotic Systems

Animal models, particularly gnotobiotic mice, offer opportunities to study archaeal colonization and host interactions under controlled conditions [28,29]. Colonization experiments with *M. smithii* have shown increased adiposity in mice, implicating archaeal metabolism in energy harvest and obesity [28]. These models may be expanded to test hypotheses about archaeal contributions to inflammatory diseases or co-infections. However, cross-species differences may limit extrapolation to humans, and standardized protocols for archaeal colonization remain underdeveloped. Furthermore, the lack of archaeal genetic tools limits mechanistic studies.

### 2.5. Bioinformatics and Systems Biology Approaches

High-throughput sequencing datasets allow for the integration of archaeal abundance and activity with broader microbiome analyses [30]. Network analyses, machine learning, and systems biology frameworks can reveal archaeal–bacterial associations and identify disease-linked microbial signatures [31]. Such approaches are particularly powerful in distinguishing correlation from potential causation, though experimental validation remains essential. Publicly available databases (e.g., Genome Taxonomy Database (GTDB) [32], SILVA [33], and RefSeq [34]) should be explicitly referenced, versioned, and updated during analysis. Furthermore, transparent reporting of computational pipelines (including software versions and parameters) is recommended.

In summary, the study of the human archaeome relies on a combination of culture-independent sequencing, specialized culturing methods, co-culture experiments, and animal models. Methodological rigor—including the use of appropriate controls, reproducible bioinformatics pipelines, and validation across multiple omics platforms—is crucial. Future progress will depend on improving archaeal cultivation, developing genetic manipulation tools, and integrating multi-omics datasets to link archaeal activity with host physiology and pathology.

## 3. Results

This review was conducted following the principles of the Preferred Reporting Items for Systematic Reviews and Meta-Analyses (PRISMA) guidelines [35].

### 3.1. Literature Search Strategy

We performed a structured search of three electronic databases—PubMed, Web of Science, and Scopus—covering the period from January 2000 to October 2025. The search was limited to peer-reviewed articles published in English. Search terms combined controlled vocabulary (MeSH terms where applicable) and free-text keywords, using Boolean operators. The core strategy included:

- (“archaea” OR “archaeome”) AND
- (“microbiome” OR “dysbiosis” OR “microbiota”) AND
- (“disease” OR “pathogenesis” OR “infection” OR “inflammation”)

Additional niche-specific terms (e.g., “oral,” “gut,” “skin,” “urogenital tract,” “cystic fibrosis,” “periodontal disease”) were added to refine the search by body site.

### 3.2. Eligibility Criteria

We included studies that met the following criteria:

1. Original research articles (experimental or observational) that investigated archaea in human-associated microbiomes.
2. Studies reporting either taxonomic characterization (16S rRNA, metagenomics, or other sequencing methods) or functional/clinical associations of archaea.
3. Both clinical and preclinical (in vitro or animal model) studies were considered if they provided mechanistic insight relevant to human health.

We excluded:

1. Studies that did not specifically analyze or report archaeal data.
2. Reviews, commentaries, conference abstracts, or case reports without primary data.
3. Articles not available in English.

### 3.3. Study Selection and Data Extraction

All search results were imported into reference management software (EndNote X9) to remove duplicates. Titles and abstracts were screened independently by two reviewers. Full-text articles were then assessed for eligibility, with disagreements resolved by discussion and consensus. Data extracted included: study design, population/sample type, methods used for archaeal detection, body site, disease context, and main findings.

### 3.4. Flow Diagram

**Figure 1:**
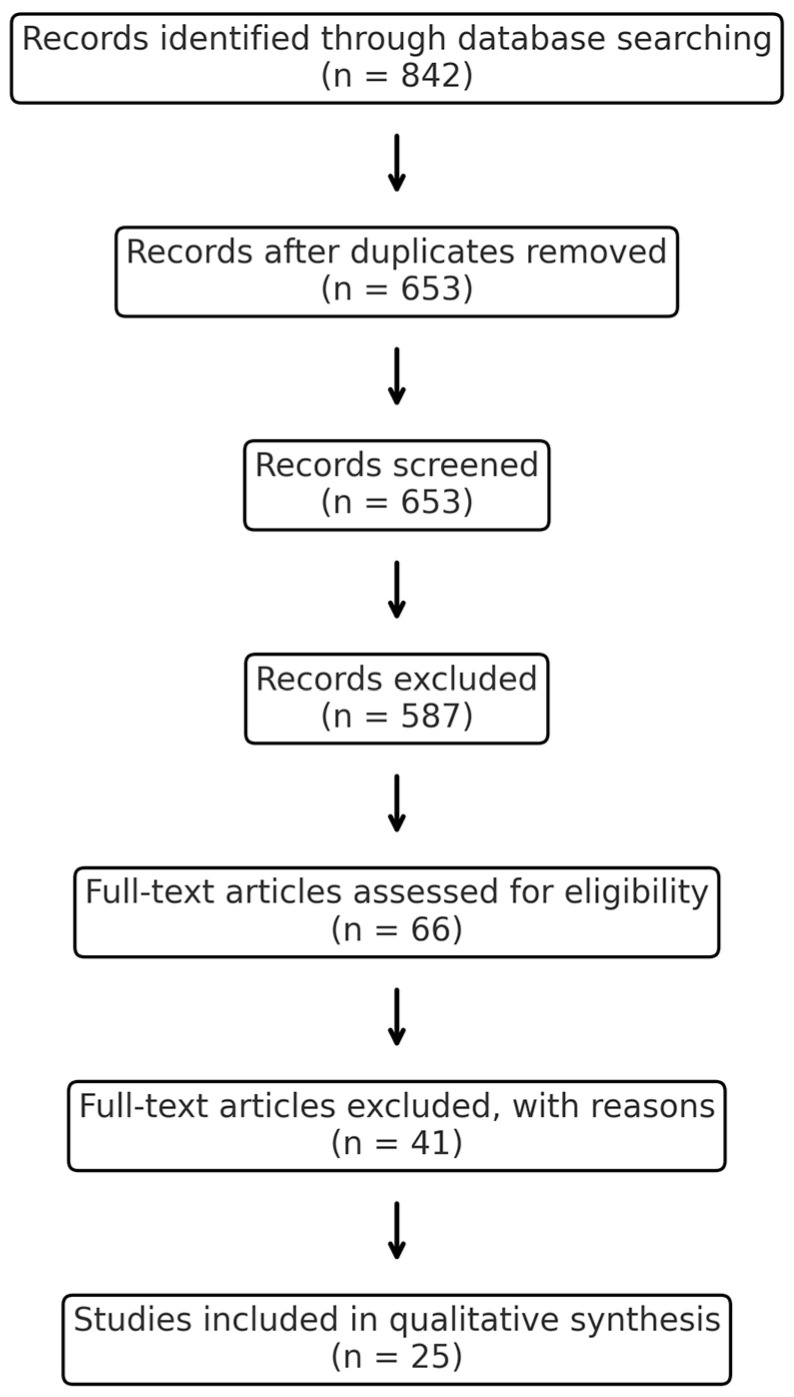
PRISMA-style flow diagram.

The selection process is summarized in a PRISMA-style flow diagram (Figure 1), showing the number of records identified, screened, assessed for eligibility, and included in the final synthesis.

PRISMA diagram showing the number of records identified, screened, assessed for eligibility, and included in the final synthesis (databases accessed on 10 June 2025).

## 4. Discussion

**Figure 2:**
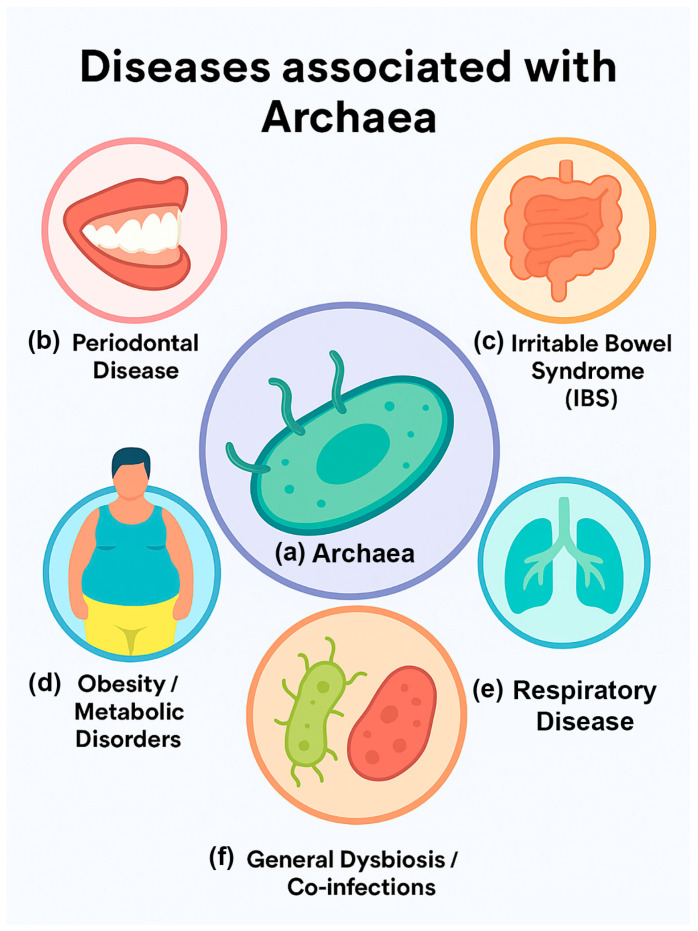
Diseases potentially associated with archaea. Schematic summary of potential disease associations of archaea, highlighting their indirect and context-dependent role in human pathology. (**a**) Archaea, represented as central elements in the microbiome, may contribute to (**b**) periodontal disease through synergistic interactions with oral bacteria, (**c**) irritable bowel syndrome (IBS) via altered fermentation and increased intestinal methane production affecting gut motility, (**d**) obesity and metabolic disorders by modulating host energy balance, (**e**) respiratory disease, such as in cystic fibrosis lungs, through persistence in polymicrobial communities where they may influence microbial ecology and inflammation, and (**f**) general dysbiosis or co-infections by acting as accessory partners that shape bacterial virulence and host responses. This schematic does not imply direct causation, but highlights hypothesized or reported associations that warrant further mechanistic investigation.

Archaea occupy a paradoxical position in human microbiology. On the one hand, they are recognized as essential members of the microbiome, especially within anaerobic environments such as the gastrointestinal tract and oral cavity [4]. On the other hand, they have never been conclusively implicated as primary pathogens in humans or other mammals [4]. The growing field of archaeal research has revealed intriguing associations between specific archaeal taxa and disease states, yet causality remains elusive. This discussion will explore three major themes: (1) the current evidence linking archaea to disease, (2) potential mechanisms of archaeal involvement in pathology, and (3) limitations and challenges in establishing archaea as pathogens (Figure 2; Table 1).

### 4.1. Evidence Linking Archaea to Disease

#### 4.1.1. Inflammatory Bowel Disease (IBD)

Several studies have reported elevated levels of methanogenic archaea in patients with Crohn’s disease or ulcerative colitis compared with healthy controls [14,27]. Among these, *Methanosphaera stadtmanae* has attracted particular attention due to its ability to elicit strong pro-inflammatory responses in vitro. For example, stimulation of human dendritic cells with *M. stadtmanae* results in robust cytokine release and activation of T cell pathways, suggesting that archaea may contribute to the heightened immune activity characteristic of IBD [16,36,49]. However, most evidence to date is based on small cohorts or in vitro studies, and direct in vivo proof of archaeal involvement remains lacking.

Despite these intriguing findings, the relationship between archaea and IBD remains inconsistent and far from conclusive. Not all studies observe archaeal enrichment in IBD cohorts, and many patients display no detectable increase in methanogen abundance [16,36]. This variability could reflect differences in geography, diet, host genetics, or even methodological limitations in archaeal detection, since many commonly used 16S rRNA primers are biased toward bacteria and miss archaeal taxa. These technical gaps highlight the importance of using archaeal-specific primers, metagenomic sequencing, and standardized protocols in future studies.

A further complication is that bacterial dysbiosis is a well-established hallmark of IBD [16,36,48]. Shifts in *Bacteroides*, *Firmicutes*, and other bacterial lineages dominate the microbiome signatures of disease, raising the question of whether archaea are primary drivers of inflammation or secondary responders to an altered gut environment. One possibility is that archaeal enrichment reflects ecological “spillover” from bacterial disruptions, in which changes in available substrates (e.g., hydrogen and acetate) provide methanogens with new growth opportunities. This scenario would position archaea more as opportunistic responders than as causal agents.

Alternatively, archaea may act as facilitators of disease progression rather than primary instigators. By consuming hydrogen and influencing fermentation pathways, methanogens can alter gut physiology, luminal pH, and gas dynamics, which in turn may affect bacterial community composition and mucosal interactions [50]. This ecological role could amplify existing dysbiosis and exacerbate inflammation without making archaea independent causes of IBD. Such indirect contributions may be subtle but clinically relevant, especially in the context of complex host–microbe interactions.

Ultimately, more work is needed to clarify whether methanogens such as *M. stadtmanae* are drivers, passengers, or modulators in the IBD disease process. Future priorities should include longitudinal studies with larger, geographically diverse cohorts, archaeal-targeted multi-omics approaches, and functional host–archaea co-culture systems. These strategies will be key in disentangling cause from correlation and defining the true significance of archaea in IBD pathogenesis.

In summary, while *M. stadtmanae* and other methanogens show intriguing associations with IBD and have demonstrated pro-inflammatory potential in vitro, the current evidence base remains inconclusive. Technical challenges in archaeal detection, small study sizes, and the dominant role of bacterial dysbiosis complicate interpretations. At present, archaea are best viewed as potential modulators or facilitators rather than established drivers of IBD, with their true role awaiting clarification through archaeal-targeted, longitudinal, and mechanistic studies.

#### 4.1.2. Periodontal Disease

The oral archaeon *Methanobrevibacter oralis* has been detected at higher prevalence in individuals with periodontitis than in healthy controls [8,36,37,38]. Several quantitative PCR and metagenomic studies suggest that archaeal load is positively correlated with disease severity, although results remain heterogeneous across populations [45,51]. Within the complex microbial biofilm of the oral cavity, methanogens may function as “helper” organisms, consuming hydrogen produced by bacterial fermentation and thereby creating favorable conditions for the growth of obligate anaerobic pathogens such as *Porphyromonas gingivalis* and *Tannerella forsythia* [8,36,37,38]. This syntrophic metabolism highlights how archaea may indirectly modulate the ecological balance of biofilms and sustain chronic inflammation.

Despite these associations, the absence of identifiable virulence factors in *M. oralis* makes it unlikely to act as a primary pathogen. Instead, its role may be more ecological, indirectly influencing disease progression by stabilizing and enriching pathogenic bacterial communities [8,36,37,38]. Moreover, periodontal disease is inherently polymicrobial and influenced by host immune responses, which complicates efforts to isolate archaeal contributions [8,36,37,38]. Another unresolved question is whether immune activation by archaeal surface structures contributes to host inflammation, as preliminary in vitro studies have suggested mild pro-inflammatory responses [48]. Clarifying these relationships will require integrated approaches that combine metagenomics, single-cell sequencing, and immune profiling in well-defined patient cohorts.

In summary, while *M. oralis* and other methanogens are consistently enriched in periodontal disease, their contribution appears to be ecological rather than directly pathogenic. By consuming hydrogen and sustaining anaerobic niches, archaea may stabilize dysbiotic bacterial biofilms that drive inflammation. However, the lack of clear virulence determinants and the complexity of host–microbiome interactions make it difficult to assign causality. Future studies integrating metagenomic, single-cell, and host immune analyses will be critical to clarify whether archaea act as passive bystanders, metabolic enablers, or active modulators of disease progression.

#### 4.1.3. Obesity and Metabolic Disorders

In gnotobiotic mouse models, colonization with *M. smithii* has been shown to increase adiposity [18,19,39]. The mechanism is thought to involve archaeal consumption of hydrogen, which lowers hydrogen partial pressure and enables bacterial fermentation to proceed more efficiently. This enhanced fermentation increases short-chain fatty acid (SCFA) production and overall energy harvest from the diet, ultimately boosting host caloric uptake [18,19,39]. More recent work in cancer has also suggested that archaeal metabolites may influence host signaling pathways, including those involved in lipid storage and glucose metabolism, expanding the potential mechanisms beyond energy harvest alone [52].

In humans, the relationship between *M. smithii* abundance and metabolic health appears more complex. Some studies have linked higher *M. smithii* levels with obesity, consistent with the hypothesis of increased energy extraction from food, while others have reported associations with leanness, possibly reflecting improved metabolic efficiency or host-microbiome adaptation to diet [53]. Additional reports suggest that archaeal abundance may correlate with dietary fiber intake or metabolic flexibility, further complicating simple associations. Still other studies have found no significant association, suggesting that archaeal effects may be modulated by host genetics, diet composition, or co-occurring bacterial taxa.

These inconsistencies underscore the context-dependent nature of archaeal–host interactions and raise the possibility that archaea may play different roles in metabolic outcomes depending on ecological and host-specific factors [53]. Longitudinal cohort studies and interventional designs, combined with multi-omics integration, will be essential to disentangle causality and determine whether archaea act as contributors, compensators, or bystanders in human obesity and related metabolic disorders.

Overall, evidence from animal models strongly supports a role for *M. smithii* in enhancing energy harvest, but human studies remain inconclusive and point to a context-dependent relationship. The variability across cohorts highlights that archaeal effects on metabolic health likely depend on host genetics, diet, and microbial community structure. Future multi-omics and intervention-based studies will be crucial to clarify whether archaea function as metabolic drivers, adaptive symbionts, or neutral commensals in obesity and related disorders.

#### 4.1.4. Other Associations

Archaea have also been detected in human niches beyond the gastrointestinal and oral environments, including the skin [9,10], respiratory tract [54], and urogenital tract [55]. On the skin, members of the class *Nitrososphaeria* are consistently found and are thought to participate in ammonia oxidation, potentially influencing local pH and the balance of bacterial communities. This ecological role raises the possibility of indirect effects on skin inflammation or dysbiosis, although direct causal links to dermatological conditions such as acne, eczema, or psoriasis remain speculative and understudied.

In the lung, archaeal DNA has occasionally been identified in both healthy individuals and patients with chronic respiratory disease [54]. Recent advances in sequencing sensitivity raise the question of whether these signals reflect true colonization, transient persistence within polymicrobial communities, or environmental contamination. Similarly, in the urogenital tract, archaeal sequences have been reported in both male and female samples [55], yet consistent findings across cohorts and functional studies are lacking, making it difficult to distinguish biological relevance from background noise. Addressing these uncertainties will require rigorous multi-site sampling, culture-based validation, and functional assays that move beyond descriptive detection.

While archaeal signatures have been identified in diverse body niches including the skin, lungs, and urogenital tract, their biological relevance remains unclear. Current data suggest primarily ecological roles, such as modulating local pH or interacting with bacterial partners, but direct links to disease are unproven. Future research combining improved detection methods with functional and mechanistic studies will be necessary to determine whether these findings reflect incidental colonization, ecological facilitation, or overlooked contributors to host physiology and pathology.

### 4.2. Potential Mechanisms of Archaeal Involvement in Disease

**Figure 3:**
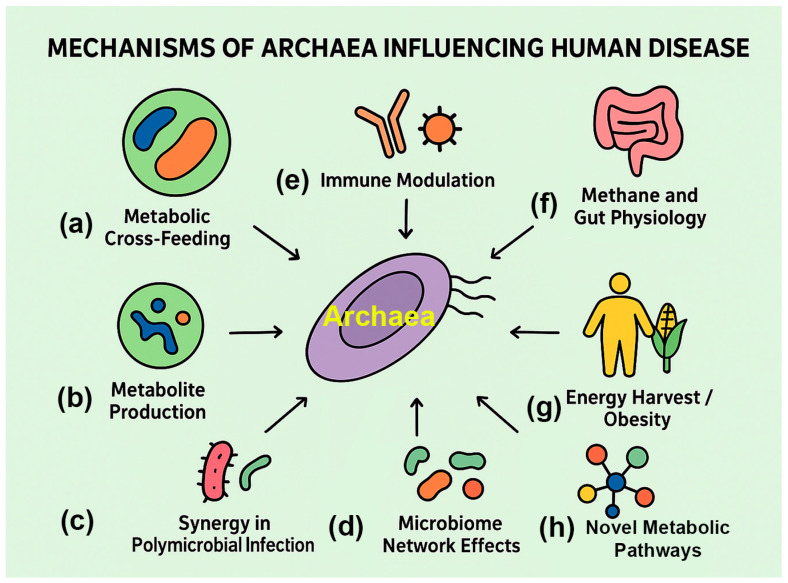
Mechanisms by which archaea may influence human disease. Schematic representation of potential pathways through which members of the human archaeome can impact host health. Central archaeal cells interact with multiple mechanisms: (**a**) Metabolic cross-feeding, where archaeal consumption of bacterial fermentation products (e.g., hydrogen) alters microbial ecosystem balance; (**b**) Metabolite production, involving unique archaeal metabolic pathways that yield novel small molecules influencing bacterial or host physiology; (**c**) Synergy and persistence in polymicrobial infection, whereby archaea may stabilize biofilms, enhance bacterial virulence, or increase community resilience in co-infection settings; (**d**) Microbiome network effects, in which archaea shape microbial community structure and inter-species interactions; (**e**) Immune modulation, reflecting archaeal antigens, extracellular vesicles, and surface molecules that may interact with host pattern-recognition receptors to trigger or dampen inflammation; (**f**) Methane and gut physiology, where methane production alters gut motility and gas balance, potentially contributing to gastrointestinal disorders; (**g**) Energy harvest/obesity, through archaeal facilitation of caloric extraction and metabolic efficiency that can promote adiposity; and (**h**) Novel or underexplored metabolic pathways, highlighting archaeal contributions to biochemical networks with potential—but still largely untested—implications for human disease.

Current evidence suggests that archaea act indirectly, shaping microbial ecology, immune signaling, and host physiology, rather than functioning like classical pathogens (Figure 3; Table 2).

#### 4.2.1. Metabolic Cross-Feeding

The best-documented role of archaea in human-associated disease contexts is through metabolic cross-feeding, a process in which archaeal metabolism indirectly shapes bacterial growth and community function. Methanogenic archaea consume molecular hydrogen generated as a byproduct of bacterial fermentation, thereby reducing hydrogen partial pressure within the local microenvironment [3]. This thermodynamically favorable process relieves feedback inhibition on bacterial fermentative pathways, enabling bacteria to extract more energy from substrates [3]. As a result, methanogenesis can act as a metabolic sink that enhances bacterial growth and survival, particularly under anaerobic conditions [3].

This ecological interaction has several implications for human disease. In the gastrointestinal tract, hydrogen consumption by methanogens such as *M. smithii* facilitates fermentation efficiency, potentially increasing caloric harvest from polysaccharides and linking archaeal activity to obesity and metabolic disorders. In periodontal disease, methanogens such as *M. oralis* thrive in inflamed, anaerobic niches of the oral cavity [8,36,37,38]. By consuming hydrogen, these archaea create conditions that favor the proliferation of anaerobic bacterial pathogens, thereby exacerbating inflammation and tissue destruction. Comparable interactions have also been suggested in respiratory and urogenital sites, though evidence remains sparse and largely descriptive. Similar dynamics may occur in polymicrobial infections elsewhere in the body, where archaeal hydrogenotrophy (i.e., attraction to hydrogen) not only relieves bacterial metabolic constraints, but may also promote biofilm stability and microbial persistence in otherwise hostile environments [8,36,37,38].

Beyond hydrogen metabolism, other archaeal metabolic products may also contribute to cross-feeding. For example, methane produced by methanogens can alter gut motility, indirectly influencing microbial colonization patterns, while archaeal production of unique metabolites such as methanol or methylamines may provide additional substrates for bacterial growth [57]. Nevertheless, many of these proposed roles are inferred from ecological models, and direct in vivo evidence for their impact on human disease remains limited. Taken together, these findings suggest that archaea may act as keystone modulators of microbial community metabolism, enhancing the ecological fitness of pathogenic bacteria and contributing to disease progression without functioning as direct pathogens themselves.

Overall, metabolic cross-feeding represents one of the clearest mechanisms by which archaea influence human health, but its consequences appear highly context dependent. While methanogens can enhance bacterial energy yield and persistence in anaerobic niches, their precise contribution to pathogenesis remains uncertain, often reflecting indirect ecological effects rather than direct virulence. Future studies employing archaeal-targeted functional assays, host–microbe co-culture systems, and longitudinal human cohorts will be critical to confirm whether archaeal cross-feeding acts primarily as a facilitator, amplifier, or bystander in disease progression.

#### 4.2.2. Immune Modulation

Archaea, though not recognized as classical pathogens, appear capable of influencing host immunity through distinctive molecular features. Several studies have highlighted that *M. stadtmanae* can activate human dendritic cells, triggering the release of pro-inflammatory cytokines such as TNF-α and IL-6, suggesting potential immunopathogenic effects even in the absence of overt infection [10,49]. Similar findings have been reported in macrophages and epithelial cells, though the strength and reproducibility of these responses vary across studies, reflecting both methodological limitations and potential inter-individual differences in immune recognition [63]. This underscores that archaeal recognition by the immune system may occur via mechanisms distinct from those established for bacteria.

Unlike bacteria, archaea lack canonical pathogen-associated molecular patterns (PAMPs) such as lipopolysaccharide (LPS) or peptidoglycan [64]. Instead, their unique structural components—including pseudomurein, ether-linked membrane lipids, and heavily glycosylated S-layer proteins—may function as immunomodulatory signals [64]. However, direct evidence for specific host receptors interacting with these molecules remains limited. Some evidence suggests they can engage pattern-recognition receptors (PRRs) such as Toll-like receptors (TLRs) or C-type lectins in noncanonical ways. For example, archaeal lipids have been hypothesized to modulate TLR2 and TLR4 pathways, potentially dampening or altering inflammatory responses compared to bacterial ligands [10,49]. These observations remain largely inferential, highlighting the need for biochemical and receptor-binding studies.

In addition to structural features, archaeal metabolic products such as methane and short-chain alcohols may indirectly influence immune tone by shaping bacterial community structure or altering epithelial barrier function [4,65]. However, it is not clear whether these effects are physiologically meaningful in vivo, as most evidence derives from correlative studies rather than mechanistic models. Furthermore, immune responses to archaea may represent evolutionary relics of ancient host–microbe interactions, given the deep evolutionary divergence of archaea from bacteria and eukaryotes [4,66]. Whether these responses confer protection, promote inflammation, or contribute to immune dysregulation in disease remains unresolved.

Immune modulation represents a plausible mechanism by which archaea influence host health, but current evidence is fragmentary and often indirect. While *M. stadtmanae* and archaeal structural components can elicit immune activation, the magnitude, context-dependence, and disease relevance of these responses remain uncertain. Clarifying whether archaeal signals act as pro-inflammatory triggers, immune dampeners, or ecological modulators will require targeted studies that directly test archaeal–host molecular interactions in both in vitro and in vivo models.

#### 4.2.3. Indirect Effects on Host Physiology

Beyond their direct interactions with bacteria, archaea can influence host physiology through metabolic activities that alter the biochemical environment of the gut and other body sites. The most widely studied example is methane production by methanogenic archaea such as *M. smithii* and *M. stadtmanae* [67]. Methane itself is metabolically inert to host cells, but it has measurable physical effects on gastrointestinal function. Clinical and experimental studies suggest that methane can slow intestinal transit by modulating smooth muscle contractility, thereby contributing to constipation-predominant irritable bowel syndrome (IBS) [40,56]. The presence of methanogens has been consistently correlated with increased breath methane levels in patients with functional gastrointestinal disorders, although causality remains debated due to variability in diagnostic methods and limited interventional data.

In the context of metabolic health, archaea also play an indirect role in regulating host energy balance. By consuming hydrogen and thereby improving the efficiency of bacterial fermentation, methanogens enhance caloric extraction from dietary polysaccharides [19,53]. This increased energy harvest has been implicated in obesity and metabolic disorders, particularly in animal models where colonization with *M. smithii* leads to greater adiposity [19,53]. In humans, however, evidence is less consistent, and effect sizes are often modest, suggesting that archaeal contributions may act as modulators rather than primary drivers of metabolic imbalance.

Archaeal contributions to host physiology may extend beyond methane and energy harvest. For example, archaeal metabolites such as methylamines have been linked to cardiovascular disease risk through their role in generating trimethylamine N-oxide (TMAO), a pro-atherogenic compound [68]. Likewise, archaeal ammonia oxidation by *Nitrososphaeria* on the skin and in the upper aerodigestive tract could influence local pH, barrier integrity, and microbial community composition, with potential downstream effects on host immune responses [69]. These pathways remain largely hypothetical in vivo, highlighting the need for direct experimental confirmation of archaeal activity in human tissues.

Although archaea lack classical virulence factors, their metabolic byproducts—including methane, methylamines, and ammonia—can indirectly modulate host physiology in ways that may contribute to gastrointestinal, metabolic, and cardiovascular disorders. Current evidence supports plausible mechanisms, but the strength, consistency, and clinical relevance of these effects remain uncertain. Future studies integrating archaeal activity into multi-omic and longitudinal microbiome models will be critical to determine whether these indirect influences represent subtle background modulators or meaningful contributors to chronic disease.

### 4.3. Barriers to Establishing Archaeal Pathogenicity

#### 4.3.1. Lack of Virulence Factors

A defining feature of established bacterial pathogens is their arsenal of virulence factors, which include protein toxins, adhesins, secretion systems, and specialized effector molecules that directly damage host tissues, evade immune defenses, or manipulate host cell signaling [70]. These features are frequently encoded on mobile genetic elements such as plasmids or pathogenicity islands, enabling rapid horizontal transfer and diversification among bacterial lineages [70]. In contrast, to date, no archaeal species associated with humans has been shown to harbor comparable systems or genetic determinants of classical virulence. Comparative genomic analyses of archaeal taxa inhabiting the human body, such as *M. smithii*, *M. stadtmanae*, and *M. oralis*, consistently reveal an absence of genes encoding classical virulence determinants [10,49].

The structural biology of archaea also argues against traditional pathogenicity. Unlike bacteria, archaea lack peptidoglycan-based cell walls and instead possess pseudomurein, proteinaceous S-layers, or unique glycosylated cell wall structures. These distinct features not only alter their interactions with the host immune system but may also insulate archaea from acquiring bacterial virulence determinants via lateral gene transfer [64]. The unusual lipid architecture of archaeal membranes—ether-linked isoprenoid chains rather than ester-linked fatty acids—further distinguishes them and may limit compatibility with bacterial pathogenicity machinery [64].

Importantly, the absence of canonical virulence factors does not mean that archaea are biologically inert. Their interactions with hosts may instead be mediated through subtler mechanisms, including metabolic cross-feeding, modulation of microbial community structure, or immune activation via unique archaeal cell envelope components [13]. Furthermore, while no toxins or secretion systems have been identified, the possibility remains that archaeal genomes encode as-yet uncharacterized proteins, small RNAs, or metabolites with immunomodulatory or cytotoxic potential [4,71]. Current functional annotations of archaeal genomes are incomplete, raising the likelihood that putative noncanonical effectors remain undiscovered. With the expansion of archaeal genomics and metagenomic studies, previously overlooked candidates for noncanonical virulence mechanisms may yet be discovered.

Taken together, the lack of known virulence determinants strongly argues against archaea acting as classical primary pathogens in mammals. Instead, they are better understood as ecological partners, metabolic modulators, or secondary contributors within polymicrobial communities. This distinction underscores the importance of reframing research questions: rather than searching for bacterial-style virulence, future studies should focus on uncovering subtle, noncanonical archaeal mechanisms that may influence host health in chronic or community-driven disease contexts.

#### 4.3.2. Methodological Challenges

The study of human-associated archaea has long been constrained by methodological limitations, resulting in their consistent underrepresentation in microbiome research [72]. Many early and even current large-scale sequencing surveys have been optimized for bacterial detection, inadvertently excluding archaea [72]. Commonly used 16S rRNA gene primers, for example, are tailored toward bacterial conserved regions and exhibit poor coverage of archaeal taxa, particularly methanogens and members of the *Nitrososphaeria* [72]. As a result, archaeal reads are often missed during amplification or discarded during bioinformatic processing pipelines, introducing a systematic bias that diminishes their apparent prevalence and ecological importance in human microbiomes [72].

Culture-based studies face even greater challenges. Standard microbial growth media, incubation conditions, and atmospheric requirements are ill-suited for archaea, many of which are strict anaerobes requiring specialized substrates (e.g., hydrogen, methanol, or formate) and long doubling times [73]. Methanogenic archaea are notoriously fastidious, often necessitating complex syntrophic relationships with bacteria in co-culture for stable growth [73]. As a result, isolation of pure archaeal strains remains rare, and the limited availability of isolates constrains functional studies that could clarify their contributions to human physiology and disease.

Even when sequencing-based approaches are employed, archaeal detection is further hindered by low abundance in mixed microbial communities and by incomplete genomic databases [74]. Many archaeal genomes remain poorly annotated, and reference collections are far less comprehensive than those for bacteria [74]. Consequently, archaeal reads are frequently misclassified or relegated to broad, uninformative taxonomic categories. This lack of taxonomic resolution creates a major barrier to associating specific archaeal species or strains with disease outcomes and limits reproducibility across studies [74].

Methodological hurdles also extend to functional characterization. Classical tools for assessing microbial virulence—such as cell culture infection models, mutagenesis systems, or animal challenge experiments—are not readily applicable to archaea [75]. Genetic manipulation remains in its infancy for most archaeal lineages, restricting the ability to directly test hypotheses about immune activation, metabolite production, or ecological roles [76]. Moreover, standard metabolomic pipelines are often not optimized for detecting unique archaeal lipids or metabolites, leaving many potential archaeal contributions invisible to current analytical workflows [76].

Recent advances provide reason for optimism. Archaeal-specific primers and improved shotgun metagenomic approaches now enable better detection and quantification of archaeal taxa [77]. Expansion of archaeal genome catalogs through initiatives such as the Human Microbiome Project and large-scale metagenomic binning has begun to fill gaps in reference databases [78]. Microfluidics, continuous-culture systems, and synthetic co-culture models also offer new avenues for maintaining and studying archaeal strains in vitro [79,80]. Moreover, advances in single-cell genomics [81,82], long-read sequencing [83,84], and high-resolution metabolomics [85] may soon allow archaeal functions to be resolved with greater precision. Nonetheless, adoption of these approaches remains uneven, and archaeal-inclusive methods have yet to be widely integrated into mainstream microbiome pipelines.

Overall, methodological constraints have created a persistent blind spot in microbiome science, where archaea are systematically underdetected and poorly characterized compared to bacteria. While emerging technologies promise to close these gaps, widespread implementation of archaeal-specific tools, improved functional assays, and richer genomic references will be essential to accurately capture their contributions to human health and disease. Addressing these challenges will not only elevate archaea from the margins of microbiome research, but also provide a more complete picture of host–microbe interactions.

#### 4.3.3. Co-Occurrence with Bacteria

A defining feature of human-associated archaea is their consistent co-occurrence with bacterial communities, particularly in disease-associated environments [12,13]. Rarely are archaea detected in isolation at pathological sites; instead, they appear embedded within polymicrobial consortia [12,13]. This recurrent pattern suggests that archaeal activity is context-dependent and shaped by microbial networks, but it complicates efforts to assign causality. It remains unresolved whether archaea act as primary contributors, synergistic partners, opportunistic colonizers, or merely incidental bystanders in disease processes [12,13].

One explanation for this pattern is ecological interdependence. Many archaea, particularly methanogens, rely on bacterial partners to supply essential substrates such as hydrogen, formate, or methyl compounds [86]. In return, archaeal consumption of these products alters the local redox balance, relieving feedback inhibition and enhancing bacterial fermentation efficiency [86]. This metabolic interlocking creates a syntrophic partnership that not only supports archaeal survival but also indirectly boosts bacterial growth [86]. In inflammatory niches such as the oral cavity or gut, archaeal–bacterial metabolic coupling may amplify dysbiosis and pathogenic processes [8,15,37]. For example, in periodontal disease, methanogens co-localize with anaerobic pathogens such as *Porphyromonas gingivalis*, where hydrogenotrophic activity by archaea may foster deeper tissue colonization and worsen inflammation [8,15,37].

Beyond metabolism, co-occurrence may influence microbial spatial organization and biofilm dynamics [38]. Archaea have been detected in structured biofilms on mucosal surfaces, where their presence could stabilize biofilm architecture or provide resilience against host defenses and antimicrobial treatments [38]. Their innate resistance to many antibiotics raises the possibility that archaea act as “community stabilizers”, buffering microbial consortia under therapeutic stress and indirectly protecting co-resident bacteria [38]. This property may complicate treatment strategies for polymicrobial infections.

From a host perspective, archaeal–bacterial co-occurrence complicates immune recognition. Bacteria provide canonical pathogen-associated molecular patterns (PAMPs) such as lipopolysaccharide, while archaea contribute distinct cell wall structures and metabolic byproducts [87]. Together, these signals may generate mixed or synergistic immune responses, potentially intensifying inflammation, modulating tolerance, or skewing immune regulation in ways not attributable to either group alone [87].

Despite these intriguing possibilities, experimental proof of archaeal–bacterial synergy in disease remains limited. The technical difficulty of isolating archaeal species and recreating their interactions with bacteria in vitro has hindered mechanistic studies [10]. Moreover, the fact that archaea are typically low-abundance members of disease-associated communities makes it challenging to separate their effects from those of dominant bacterial taxa [10]. Future studies integrating spatial transcriptomics, metabolomics, and synthetic co-culture systems will be critical to determine whether archaeal co-occurrence is mechanistically linked to disease or simply reflects shared ecological niches.

Overall, archaeal–bacterial co-occurrence represents a recurring yet poorly understood feature of human dysbiosis. While current evidence supports the idea that archaea shape bacterial metabolism, biofilm resilience, and host immune responses, definitive proof of causality is lacking. Recognizing and dissecting these cross-domain interactions will be essential to determine whether archaea act as silent enablers of bacterial pathogenesis or as overlooked modulators of microbial ecology with significant clinical implications.

#### 4.3.4. Limited Genetic Tools

One of the greatest obstacles in deciphering the role of archaea in human health and disease is the paucity of reliable genetic tools. In bacterial pathogenesis research, genetic manipulation—via knockouts, knockdowns, overexpression systems, or CRISPR-based editing—has been central to establishing causal links between candidate genes and virulence traits [88]. In contrast, archaeal systems remain far more difficult to manipulate, creating a major barrier to mechanistic studies of their physiology, host interactions, and potential contributions to pathology [73].

Several factors contribute to this challenge. First, archaea possess highly distinctive cellular machinery that differs substantially from both bacteria and eukaryotes. Their DNA replication, transcription, and translation systems are a hybrid of bacterial and eukaryotic features, complicating the transfer of molecular biology techniques developed in model organisms [89]. Second, archaeal cell envelopes—often composed of pseudomurein or heavily glycosylated S-layer proteins—pose barriers to DNA uptake, reducing the efficiency of transformation methods [90]. Third, archaeal extremophily and strict metabolic requirements make standard laboratory protocols inadequate, as many species demand specialized growth conditions (e.g., strict anaerobiosis, high temperatures, or unusual substrates) that are difficult to reproduce consistently [73,91].

While genetic systems have been developed for a handful of archaeal species, such as *Haloferax volcanii* [80] and *Methanosarcina acetivorans* [92], these are not the dominant taxa found in human-associated microbiomes. The clinically relevant methanogens, such as *M. smithii*, *M. stadtmanae*, and *M. oralis*, remain refractory to routine genetic manipulation [73]. This limits our ability to experimentally test hypotheses about archaeal biology, such as whether archaeal cell wall structures act as immune activators or whether archaeal metabolites directly shape bacterial virulence. As a result, functional roles remain inferred rather than demonstrated.

This lack of genetic tractability creates a vicious cycle: without experimental validation, archaeal genes remain annotated largely as “hypothetical proteins,” and without functional annotation, the incentive to develop species-specific genetic tools remains low. This self-reinforcing gap leaves archaeal contributions to human disease defined primarily by correlative studies—such as co-occurrence networks, metagenomic associations, and breath methane diagnostics—rather than rigorous tests of causality.

The emergence of new molecular technologies, however, offers hope. Advances in metagenomics [93], metatranscriptomics [94], and metaproteomics [95] are beginning to shed light on archaeal activity in situ, while synthetic biology and cell-free expression systems may provide alternative routes to probe archaeal protein function without direct genetic manipulation. Furthermore, because many CRISPR–Cas systems are archaeal in origin, they may eventually be adapted into archaeal-specific genome editing platforms, provided technical hurdles such as transformation efficiency and host defense systems can be overcome [96].

In sum, the limited availability of genetic tools remains one of the most pressing barriers to advancing archaeal biology in the context of human health. Without tractable systems, the functional roles of archaeal genes and pathways will remain speculative, impeding progress toward causal models of archaeal–host and archaeal–bacterial interactions. Expanding archaeal-specific molecular methods, including genome editing and expression systems, will be essential for moving beyond correlation and unlocking the mechanistic basis of archaeal contributions to disease.

### 4.4. Future Insights from Spatial Transcriptomics

**Figure 4:**
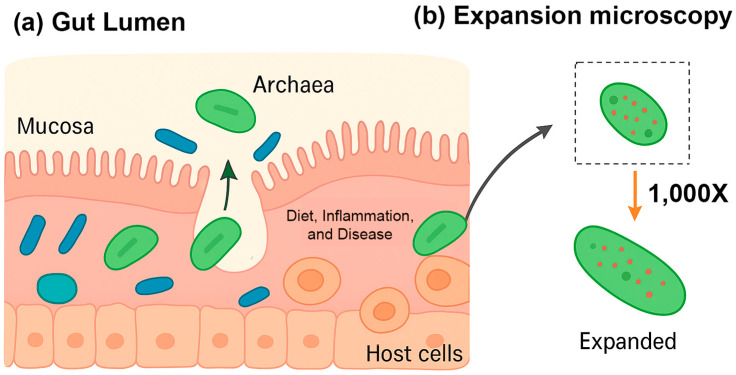
Spatial mapping of archaea in the human gut using bacterial-MERFISH. This schematic illustrates how highly multiplexed spatial transcriptomics reveals the behavior and interactions of gut archaea in situ. (**a**) Archaea (green) occupy diverse microniches along the gut lumen and mucosa, each defined by varying oxygen levels, nutrient availability, and proximity to host cells (orange). Methanogenic archaea, such as *M. smithii*, are shown metabolically coupled to neighboring hydrogen-producing bacteria (blue), converting H_2_ to methane. The technique simultaneously profiles host transcripts, allowing visualization of host–archaea interactions, including modulation of genes involved in immunity, mucus secretion, and barrier function. Changes in archaeal gene expression in response to diet, inflammation, or disease states (e.g., IBD) are indicated by variable shading. Bacterial-MERFISH overcomes technical limitations by enlarging microbial cells via expansion microscopy and profiling unculturable species directly in their native environment. This approach enables the dissection of archaeal contributions to gut metabolism, host physiology, and disease processes. (**b**) In expansion microscopy, cells are enlarged up to 1000-fold, making intracellular RNAs resolvable with conventional microscopes.

Recent advances in highly multiplexed spatial transcriptomics, notably the bacterial-MERFISH technique developed in the Moffitt lab, are transforming our understanding of archaea in human health [97] (Figure 4a). Unlike traditional sequencing methods, which dissociate microbes from their environment, bacterial-MERFISH preserves the spatial context of microbial cells while profiling gene expression at high resolution [97]. This enables researchers to observe not only which genes are active in archaea, but also precisely where and in association with which other cells this activity occurs. Such insights are particularly relevant in the gut, where archaea occupy diverse microenvironments and engage in complex interactions with both bacteria and host tissues.

The gut is a heterogeneous ecosystem, with microniches defined by gradients in oxygen, nutrient availability, and proximity to host tissue [6,13]. By maintaining spatial context, bacterial-MERFISH allows for the identification of archaea within these niches and the characterization of their gene expression in response to local conditions. Most gut archaea are methanogens, such as *M. smithii*, which consume hydrogen (H_2_) produced by neighboring bacteria and convert it into methane [6,13]. Spatial transcriptomics can map the co-localized gene expression of methanogens and hydrogen-producing bacteria, providing a detailed view of these metabolic partnerships and revealing how they vary across different regions of the gut.

Bacterial-MERFISH can simultaneously profile microbial and host transcripts, offering an unprecedented window into host–microbe communication [97]. This capability allows researchers to map how the presence of archaea influences host gene expression, including genes involved in immunity, mucus production, and barrier function. It also enables the testing of the “archaebiotics” concept, whereby certain archaea may act as beneficial microbes to support mucosal health or enhance resistance to infection. By visualizing these interactions in situ, bacterial-MERFISH can provide direct evidence of beneficial host–archaea dialogues [97].

Archaeal activity is sensitive to local nutrient availability and environmental cues [13]. Spatial transcriptomics can reveal how archaea modulate their gene expression in response to dietary shifts, host-derived polysaccharides, or inflammatory signals, as observed in conditions such as inflammatory bowel disease (IBD) [5]. This approach also allows the dissection of disease heterogeneity, uncovering differences in archaeal activity along the gut axis or between individuals with varying disease manifestations.

Although archaea are generally non-pathogenic, as noted above, associations with chronic conditions—including metabolic and cardiovascular diseases—have been reported [13]. Bacterial-MERFISH can clarify these relationships by linking archaeal activity in situ with local host responses. By integrating spatial maps of microbial and host gene expression, researchers can gain mechanistic insight into the debated roles of methanogens in disease, distinguishing direct metabolic effects from secondary or opportunistic interactions.

Two key hurdles have historically limited studies of archaea: their small size and difficulty in cultivation. Bacterial-MERFISH overcomes both. Using expansion microscopy, cells are enlarged up to 1000-fold, making intracellular RNAs resolvable with conventional microscopes (Figure 4b). The in situ nature of the technique also bypasses cultivation bias, enabling the profiling of unculturable archaea directly within their native microenvironments. These methodological advances open new avenues for understanding the contributions of archaea to gut health, host metabolism, and disease pathogenesis.

In summary, spatial transcriptomics provides an unprecedented lens to study archaea in their native context, offering mechanistic insights into their metabolic partnerships, host interactions, and potential roles in health and disease. This approach represents a crucial step toward defining archaea not just as passive residents of the gut but as active modulators of microbial ecosystems and human physiology.

### 4.5. Conceptual Implications

The accumulated evidence indicates that archaea should be viewed not as classical pathogens but as “accessory microbes” whose influence in human disease is often indirect and context dependent. Rather than deploying virulence factors that directly damage host tissues, archaea appear to act through subtler ecological and metabolic mechanisms—such as hydrogen consumption, methane production, or modulation of microbial networks—that can shift community balance and alter host physiology. In this sense, their role is more analogous to commensals that become opportunistic under specific environmental pressures, or to ecological facilitators that enhance the activity of co-resident bacteria.

Importantly, describing archaea as accessory does not diminish their clinical significance. On the contrary, their capacity to amplify bacterial pathogenicity, alter host immune tone, or stabilize disease-associated biofilms suggests that small changes in archaeal abundance or activity could disproportionately impact disease trajectories. For instance, methanogens may promote obesity by enhancing caloric harvest, exacerbate periodontal disease by supporting anaerobic pathogens, or influence gastrointestinal disorders through methane-mediated changes in motility. Such effects highlight that archaea may act as hidden drivers of disease ecology, shaping outcomes without being the primary infectious agent.

Conceptually, this perspective challenges the traditional pathogen–commensal dichotomy. Archaea occupy a “gray zone” where their contribution to pathology is conditional, emergent from cross-domain interactions and metabolic interdependencies. This raises important theoretical questions: Should definitions of pathogenicity expand to include indirect ecological facilitation? How can we disentangle causality when the disease-relevant unit may be the microbial consortium rather than a single taxon? Addressing these questions will require methodological innovation as well as a conceptual reframing of how archaeal functions are incorporated into models of dysbiosis.

Overall, the conceptual implications of the current evidence are clear: archaea must be recognized as integral, if often indirect, participants in human disease ecology. Future studies should prioritize moving from correlation to causation by combining multi-omics approaches, archaeal-inclusive experimental models, and ecological theory. Only by embracing this broader framework can we determine whether archaea are silent enablers of bacterial pathogenesis, overlooked modulators of host physiology, or both—roles that may ultimately redefine our understanding of the human microbiome in health and disease.

### 4.6. Evolutionary Perspectives

**Figure 5:**
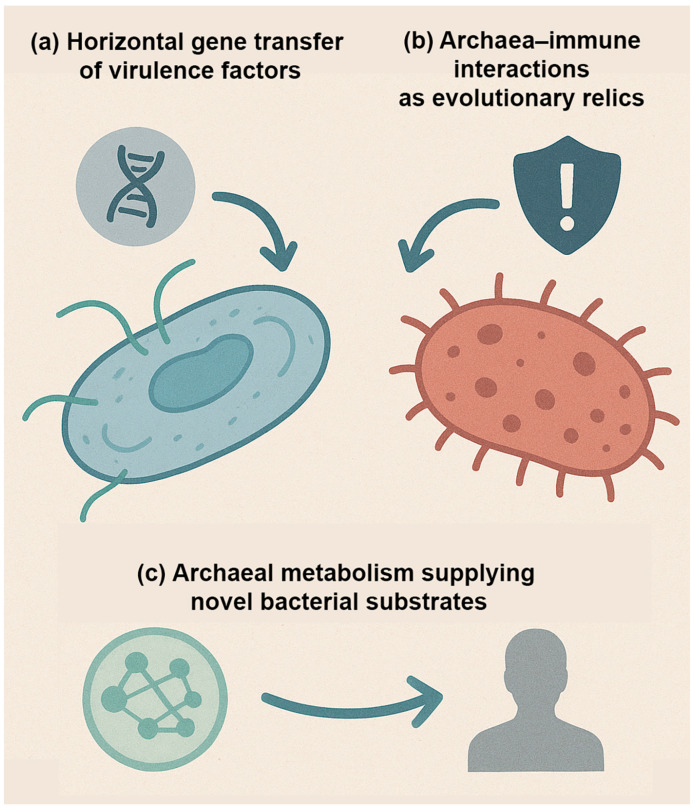
Overlooked contributors to pathology: potential roles of archaea in human disease. Archaea may influence pathology through (**a**) horizontal gene transfer of virulence factors to bacteria, (**b**) immune interactions representing evolutionary relics of early host–microbe relationships, and (**c**) metabolic cross-feeding, where archaeal products such as methane reshape bacterial communities and host physiology. Though not established pathogens, archaea may act as hidden modulators of disease processes.

Archaea occupy a unique position in the tree of life, sharing features with both bacteria and eukaryotes yet remaining fundamentally distinct. Their unusual biology and long evolutionary history raise intriguing questions about their potential role in human disease, not only in the present, but also in the future [4]. Unlike bacteria, which have repeatedly evolved pathogenic strategies across diverse lineages, archaea have not yet produced definitive pathogens. However, reviewers have noted that this absence should not be interpreted as evidence of evolutionary incapacity. Instead, it reflects the current limits of sampling, cultivation, and clinical recognition of archaeal contributions to disease. Nevertheless, considering their deep evolutionary roots and capacity for genetic and ecological innovation, several possibilities warrant exploration (Figure 5).

First, the potential for horizontal gene transfer (HGT) must be considered (Figure 5a). Although archaea appear more insulated from bacterial gene exchange due to differences in cell envelope composition, plasmids and mobile elements are widespread in archaeal lineages [98]. Experimental studies and metagenomic analyses have shown that gene flow between archaea and bacteria is possible, especially in shared niches where syntrophic interactions occur [99]. It remains conceivable that under the right selective pressures, archaeal species could acquire bacterial virulence determinants—such as adhesins, proteases, or toxin-like proteins—and repurpose them for survival in mammalian hosts [99]. While no such event has been documented, the evolutionary potential should not be dismissed, particularly given the dynamic nature of microbial ecosystems within the human body.

Second, archaeal interactions with the immune system may represent evolutionary relics of ancient host–microbe encounters (Figure 5b). Archaea are thought to have coexisted with early eukaryotic ancestors and may have played a role in shaping the evolution of innate immune recognition systems [4]. For instance, mammalian pattern recognition receptors (PRRs) evolved to detect conserved microbial molecules, many of which are absent or altered in archaea [4]. This raises the possibility that immune responses to archaeal components, such as pseudomurein or unique glycoproteins, may reflect vestiges of interactions with extinct microbial lineages [4]. These vestigial interactions may partially explain why some archaeal molecules provoke inflammatory responses in the absence of canonical bacterial PAMPs, and why archaeal signals can synergize with bacterial stimuli to intensify or dampen host pathology.

Third, archaea may serve as reservoirs of novel metabolic pathways that influence human disease in unanticipated ways (Figure 5c). Their capacity for methanogenesis, ammonia oxidation, and other specialized chemistries introduces metabolic functions not typically found in bacteria [12]. These pathways can reshape microbial community dynamics and host physiology by altering hydrogen balance, methane levels, nitrogen flux, and short-chain fatty acid availability [12]. As new archaeal lineages are discovered in the human microbiome—including skin- and airway-associated *Thaumarchaeota* (*Nitrososphaeria*)—the potential for unconventional metabolic contributions to disease becomes increasingly plausible [13]. Archaea may not act as pathogens in the classical sense, but their enzymatic repertoire could indirectly shape pathological processes by sustaining dysbiosis or fueling bacterial pathogenicity. Future work will need to disentangle whether these contributions represent adaptive traits shaped by host association or collateral byproducts of archaeal survival strategies in complex ecosystems.

Addressing these evolutionary questions requires interdisciplinary collaboration. Microbiologists can uncover archaeal diversity and physiology through cultivation and -omics approaches, while evolutionary biologists can trace the origins of archaeal traits and their potential for gene exchange. Clinicians, in turn, can provide insights into how archaeal presence correlates with disease states and patient outcomes.

Taken together, evolutionary perspectives highlight the paradox of archaea as ancient yet understudied members of the human microbiome. While they have not yet evolved classical pathogenic lineages, their ability to exchange genes, interact with host immunity, and introduce unique metabolic functions suggests that they could influence human health in subtle but consequential ways. Rather than being dismissed as evolutionary dead-ends, archaea should be viewed as latent reservoirs of genetic and metabolic potential whose relevance to human disease may become more apparent as new tools and models are developed.

### 4.7. Ethical and Practical Considerations

As archaeal research advances toward translational applications, ethical and practical considerations must be placed at the forefront. Unlike bacterial therapeutics, where decades of clinical experience provide a framework for safety evaluation, archaeal interventions remain largely uncharted territory. Manipulating the human archaeome—whether through archaeal probiotics, targeted antimicrobials, or engineered consortia—carries the risk of unintended ecological and physiological consequences [13]. Archaea are deeply integrated into host-associated microbial networks, and perturbations could destabilize these ecosystems in ways that are difficult to predict. Even apparently subtle interventions, such as altering methanogen abundance, may have cascading effects on bacterial community dynamics, gas metabolism, and immune homeostasis.

One major challenge lies in risk assessment. Because archaea are not known pathogens, there may be a tendency to assume they are inherently benign. However, as evidence grows for their roles in metabolic cross-feeding, immune modulation, and dysbiosis, therapeutic alteration of archaeal abundance or activity could inadvertently worsen disease states [4,10]. This underscores the need for standardized risk–benefit analyses that account not only for short-term clinical outcomes but also for longer-term ecological stability. Longitudinal and multi-generational microbiome monitoring will be critical to identify delayed effects that might otherwise be overlooked.

Another consideration is the ecological spillover of archaeal interventions. Engineered archaea or archaeal-targeting therapies could spread beyond the individual host into environmental reservoirs, with unknown consequences for global microbial communities [100]. Given the resilience and adaptability of archaea, release into natural ecosystems may have profound ecological effects that warrant pre-emptive safeguards. Practical strategies could include biocontainment systems, kill-switch mechanisms, or strict environmental monitoring to minimize unintended dissemination.

Ethical questions also arise around dual-use research. As tools for archaeal genetic manipulation improve, there is a theoretical risk of creating pathogenic strains, either inadvertently or deliberately. While such risks remain speculative, governance frameworks must anticipate potential misuse, just as they have for bacterial and viral genetic engineering. Ethical oversight bodies will need to adapt existing guidelines for synthetic biology to explicitly address archaeal research, balancing innovation with security.

From a practical standpoint, the development of archaeal diagnostics and therapeutics will require interdisciplinary oversight. Regulatory agencies, bioethicists, clinicians, and microbiologists must work together to ensure that novel interventions are evaluated not only for efficacy, but also for societal and ecological safety. Public communication will also be critical: framing archaea as “hidden partners” rather than “unknown pathogens” may help prevent misperceptions and foster informed dialogue about the risks and benefits [4,10]. Importantly, community engagement and public trust will determine whether archaeal interventions are embraced or resisted in clinical practice.

In summary, as archaeal biology transitions from basic science to clinical application, the field must adopt a precautionary yet proactive stance. Ethical foresight, robust safety frameworks, and transparent communication will be essential to ensure that the integration of the archaeome into medical practice benefits human health without compromising ecological or societal stability. Ultimately, the promise of archaeal-based interventions can only be realized if their development is guided by a commitment to responsible innovation, long-term monitoring, and global stewardship of microbial diversity.

## 5. Conclusions

The study of the human archaeome is still in its infancy, yet the evidence accumulated to date suggests that archaea are neither trivial bystanders nor classical pathogens. Instead, they occupy a gray area in which their contributions to health and disease are subtle, context-dependent, and mediated largely through interactions with bacterial partners and host physiology. This complexity makes them both fascinating and challenging to study.

Future research should prioritize the systematic expansion of archaeal diversity catalogs across multiple body sites, including underexplored niches such as the skin, respiratory tract, and urogenital tract [4,10]. Advances in sequencing technologies and archaeal-specific primers are beginning to uncover lineages, such as *Nitrososphaeria*, that were long overlooked by bacterial-centric approaches [4,9]. However, mere detection is insufficient: robust culture methods and genetic tools will be essential to bring these organisms into the laboratory, where their functions can be interrogated in controlled systems. Spatial resolution will also be critical; adapting high-resolution technologies such as bacteria-MERFISH—already transforming bacterial microbiome studies [97]—may allow archaeal taxa to be visualized in situ, mapped within host tissues, and studied in direct spatial relation to bacterial partners.

Equally important are functional studies in host contexts, ranging from gnotobiotic animal models to organoid co-cultures, which can provide direct evidence of how archaea modulate immune responses, alter gut motility, or interact with bacterial pathogens [28,29]. These approaches will help clarify whether archaeal associations with disease—such as inflammatory bowel disease, obesity, and periodontitis—reflect causality, opportunism, or ecological side effects of dysbiosis.

Another priority is deciphering archaeal–bacterial synergy, which may represent the most clinically relevant axis of archaeal influence. Metabolic cross-feeding, methane-mediated changes in gut physiology, and co-structuring of biofilms are all mechanisms by which archaea may amplify bacterial virulence or persistence [38,58,79]. Parsing these relationships will require integrated ecological, genomic, and immunological perspectives. Spatial multi-omics approaches that combine bacteria MERFISH [97], metabolite imaging, and single-cell sequencing could provide the resolution needed to track these interactions at the host–microbe interface, opening the way for therapeutic targeting of archaeal–bacterial consortia.

Clinical translation represents the next frontier. Archaeal biomarkers—whether based on abundance, metabolic activity, or immune signatures—could enhance diagnostic accuracy in diseases marked by dysbiosis. Microbiome engineering strategies, including archaeal-targeted antimicrobials, archaeal probiotics, or engineered cross-domain consortia, may eventually be harnessed to restore balance in disrupted microbial ecosystems. Moreover, archaeal metabolic pathways could be exploited as novel drug targets or leveraged in precision medicine to modulate host physiology [17,67,87]. At the same time, ethical and safety considerations must guide translation, given the unpredictability of intervening in systems that have co-evolved with humans for millennia [100].

Ultimately, the central challenge is to shift from descriptive catalogs to actionable insight. Rather than asking whether archaea are pathogens, future work must define the continuum of roles they occupy—from benign commensals and ecological stabilizers to opportunistic facilitators of disease. By combining cutting-edge discovery tools with translational innovation, the archaeome can be repositioned not only as a subject of basic science curiosity, but as a potential clinical lever for diagnostics, therapeutic development, and microbiome engineering.

## Figures and Tables

**Table 1:** Archaeal taxa associated with human diseases and conditions.

Disease/Condition	Archaeal Taxa Involved	Proposed Role/Mechanism	Evidence Strength	References (PMIDs)
Inflammatory Bowel Disease (IBD)	*Methanosphaera stadtmanae*, *Methanobrevibacter smithii*	Induces pro-inflammatory responses; increased abundance in some IBD patients	Moderate	[16,36]
Periodontal Disease	*Methanobrevibacter oralis*	Hydrogen consumption supports anaerobic bacterial growth in oral biofilms	Strong association, unclear causality	[8,36,37,38]
Obesity/Metabolic Disorders	*Methanobrevibacter smithii*	Enhances bacterial fermentation efficiency; promotes increased host energy harvest	Moderate (mouse models, variable human data)	[18,19,39]
Irritable Bowel Syndrome (IBS)	Methanogens (general)	Methane linked to slowed intestinal motility; may exacerbate constipation-type IBS	Moderate	[40,41]
Colorectal Cancer	*Methanobrevibacter smithii*, other methanogens	Detected at higher abundance in some tumor microbiome studies; role unclear	Weak/Correlative	[42,43]
Respiratory Disease (e.g., cystic fibrosis lungs)	*Methanobrevibacter* spp.	Found alongside bacterial pathogens in lung samples; ecological rather than causal role	Weak/Incidental	[44,45]
General Dysbiosis/Co-infections	Multiple methanogens	Synergistic effects with bacterial pathogens via metabolic cross-feeding	Strong concept, limited direct proof	[46,47,48]

**Table 2:** Proposed mechanisms by which archaea may influence human health and disease.

Mechanism	Archaeal Taxa Involved	Description	Evidence Type	Key References (PMIDs)
Metabolic Cross-Feeding	*Methanobrevibacter smithii*, *Methanobrevibacter oralis*	Consumption of hydrogen by methanogens enhances bacterial fermentation, supporting pathogen growth	Co-culture, microbiome studies	[3,7,8]
Immune Modulation	*Methanosphaera stadtmanae*	Activation of dendritic cells and cytokine production; unique archaeal cell wall components may act as immune triggers	In vitro immunology	[4,49]
Methane and Gut Physiology	General methanogens	Methane slows intestinal motility, potentially contributing to constipation and IBS	Breath methane studies, animal models	[40,56]
Energy Harvest/Obesity	*Methanobrevibacter smithii*	Hydrogen removal improves bacterial breakdown of polysaccharides, increasing caloric extraction	Gnotobiotic mouse models, human studies	[18,19,39,53,57]
Synergy in Polymicrobial Infection	*Methanobrevibacter oralis*, others	Presence of archaea creates favorable niches for anaerobic pathogens (e.g., periodontal bacteria)	Oral biofilm studies	[7,8,58]
Metabolite Production	Multiple methanogens	Methane and ammonia production may alter mucosal environment and host tissue function	Metabolomics, breath tests	[9,59]
Microbiome Network Effects	Multiple lineages	Archaea may act as keystone species influencing bacterial community composition and stability	Systems biology, network modeling	[60,61,62]

## Abbreviations

The following abbreviations are used in this manuscript:

LGT	Lower gastrointestinal tract
UAT	Upper aerodigestive tract
IBS	Irritable bowel syndrome
IBD	Inflammatory bowel disease
SCFA	Short-chain fatty acid
TNF-alpha	Tumor necrosis factor alpha
IL-6	Interleukin 6
LPS	lipopolysaccharide
PAMPS	Pathogen-associated molecular patterns
TLR-2,4	Toll-like receptor-2,4
TMAO	Trimethylamine N-oxide
MERFISH	Multiplexed error-robust fluorescence in situ hybridization
PRRs	Pattern-recognition receptors
FMT	Fecal microbiota transplant
